# Supplementary material for: Influence of sociodemographics on human mobility
Source: Sci Rep. 2015 May 20;5:10075. doi: 10.1038/srep10075 (PMC4438721; doi:10.1038/srep10075)
Supplement: Supplementary Information [file srep10075-s1.pdf]

Supporting Information: Influence of sociodemographic characteristics on human mobility

Maxime Lenormand[1], Thomas Louail[2,3], Oliva G. Cantú-Ros[4], Miguel Picornell[4], Ricardo Herranz[4], Juan Murillo Arias[5], Marc Barthelemy[2,6], Maxi San Miguel[1], and José Javier Ramasco[1]

[1]IFISC, Instituto de Física Interdisciplinar y Sistemas Complejos (CSIC-UIB), Campus Universitat de les Illes Balears, E-07122 Palma de Mallorca, Spain
[2]Institut de Physique Théorique, CEA-CNRS (URA 2306), F-91191, Gif-sur-Yvette, France
[3]Géographie-Cités, CNRS-Paris 1-Paris 7 (UMR 8504), 13 rue du four, FR-75006 Paris, France
[4]Nommon Solutions and Technologies, Plaza del Marqués de Salamanca 11, 28006 Madrid, Spain
[5]BBVA Data & Analytics, Avenida de Burgos 16D, 28036 Madrid, Spain
[6]Centre d'Analyse et de Mathématique Sociales, EHESS-CNRS (UMR 8557), 190-198 avenue de France, FR-75013 Paris, France

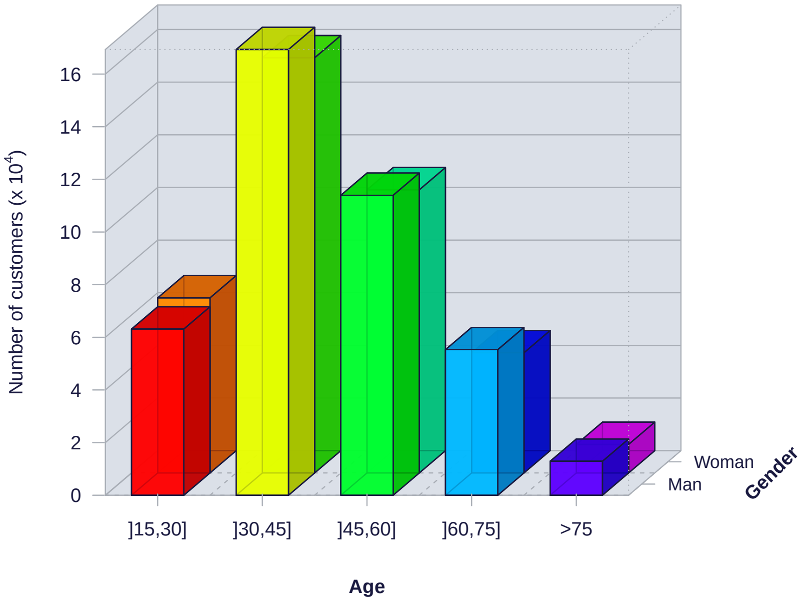

Figure S1: **Histogram of the joint distribution of individuals according to the gender and the age.**

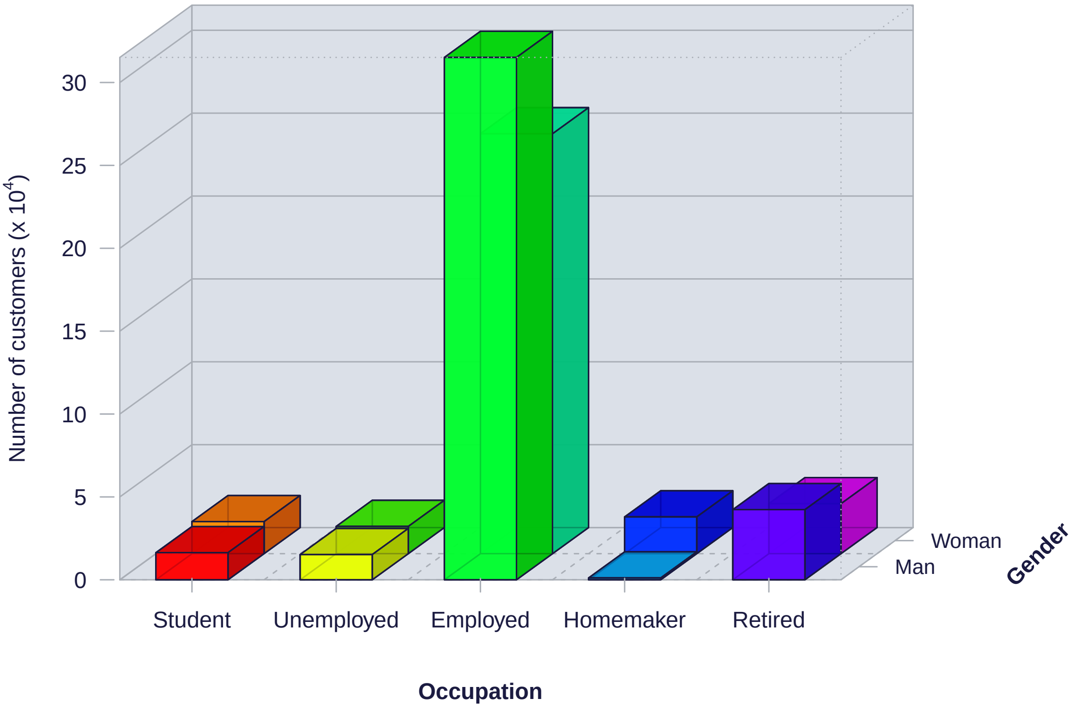

Figure S2: **Histogram of the joint distribution of individuals according to the gender and the occupation.**

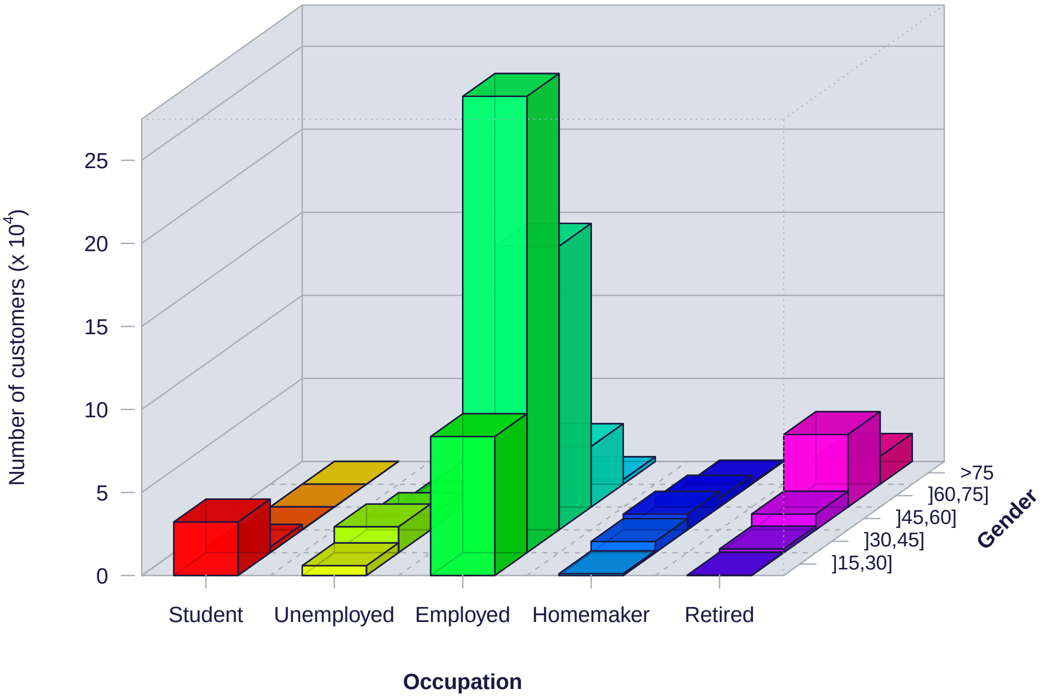

Figure S3: **Histogram of the joint distribution of individuals according to the age and the occupation.**

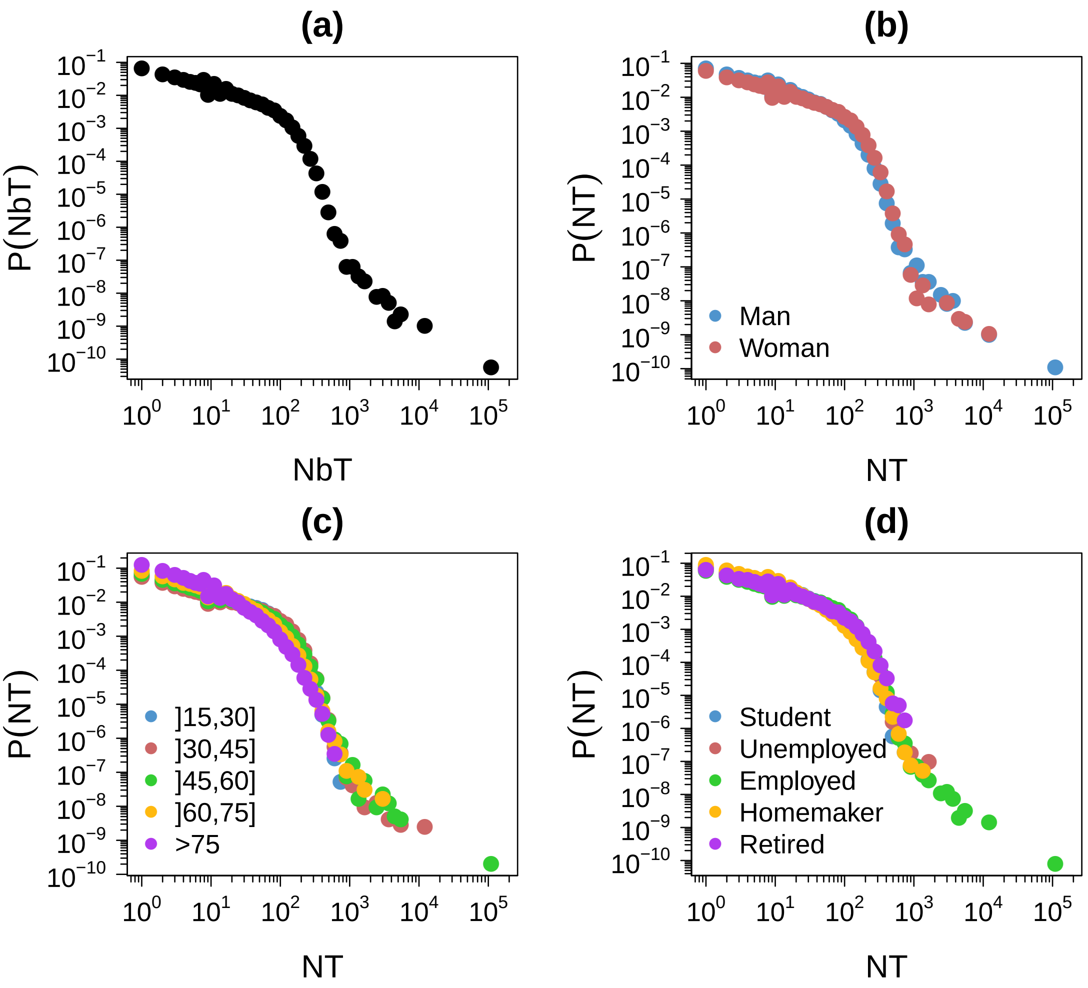

Figure S4: **Probability density function of the number of transactions per individual (a), according to the gender (b), the age (c) and the occupation (d).**

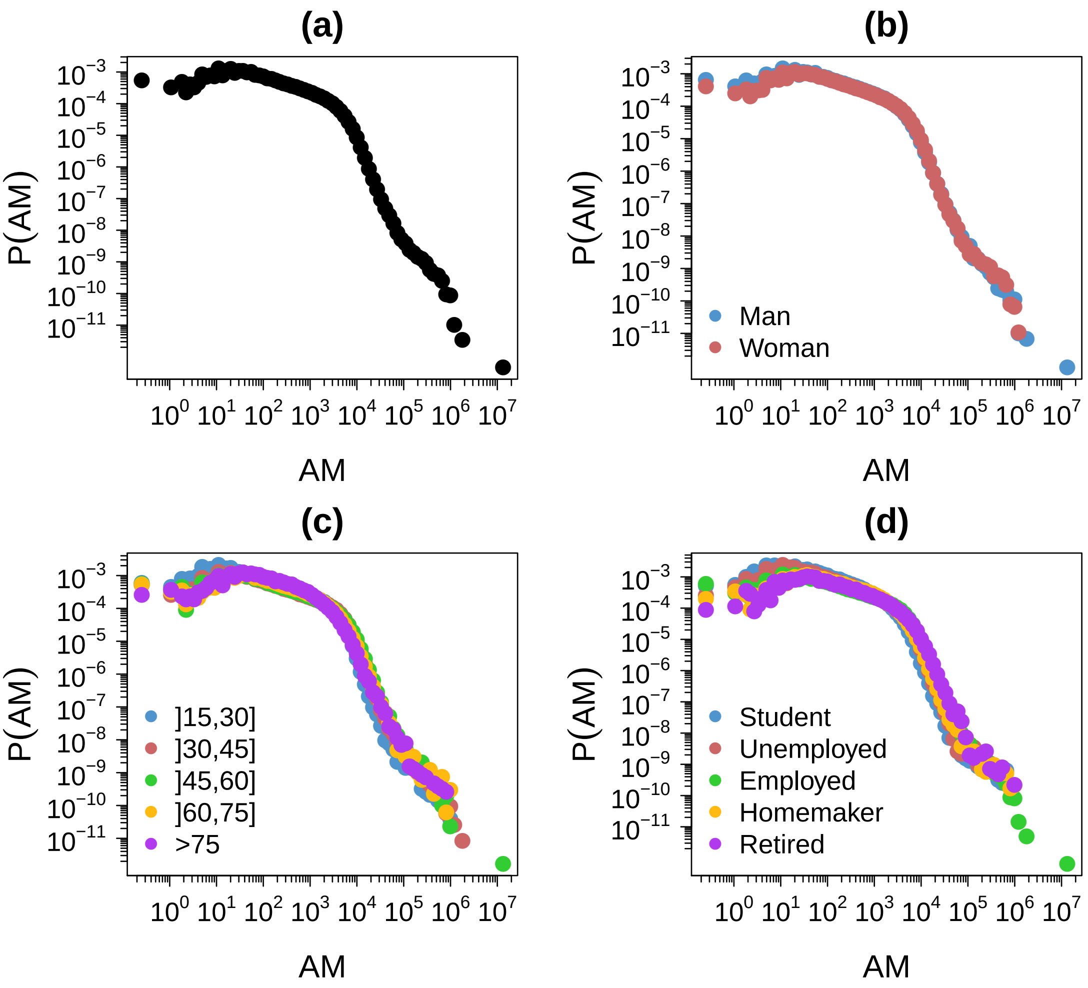

Figure S5: **Probability density function of the amount of money spent in 2011 per individual (a), according to the gender (b), the age (c) and the occupation (d).**

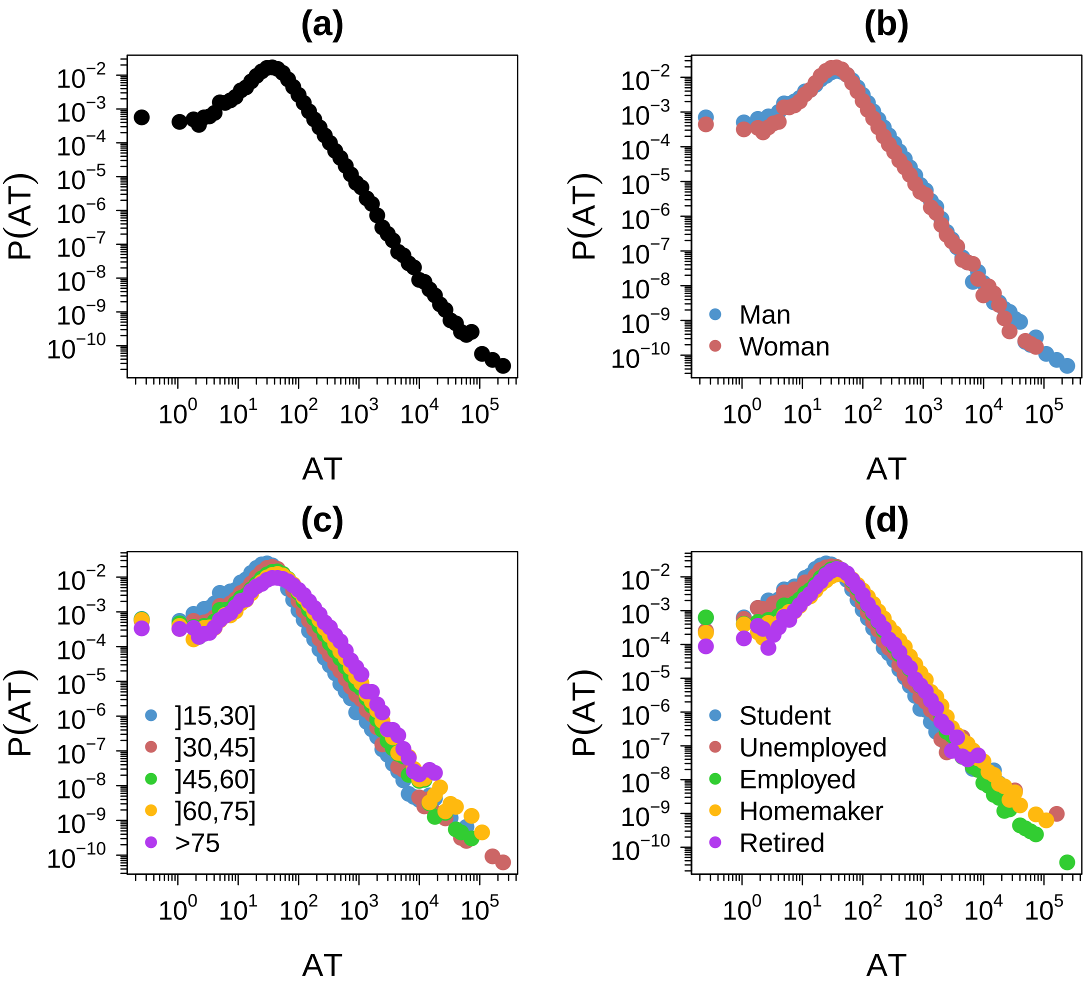

Figure S6: **Probability density function of the average amount of money spent per transaction and per individual (a), according to the gender (b), the age (c) and the occupation (d).**

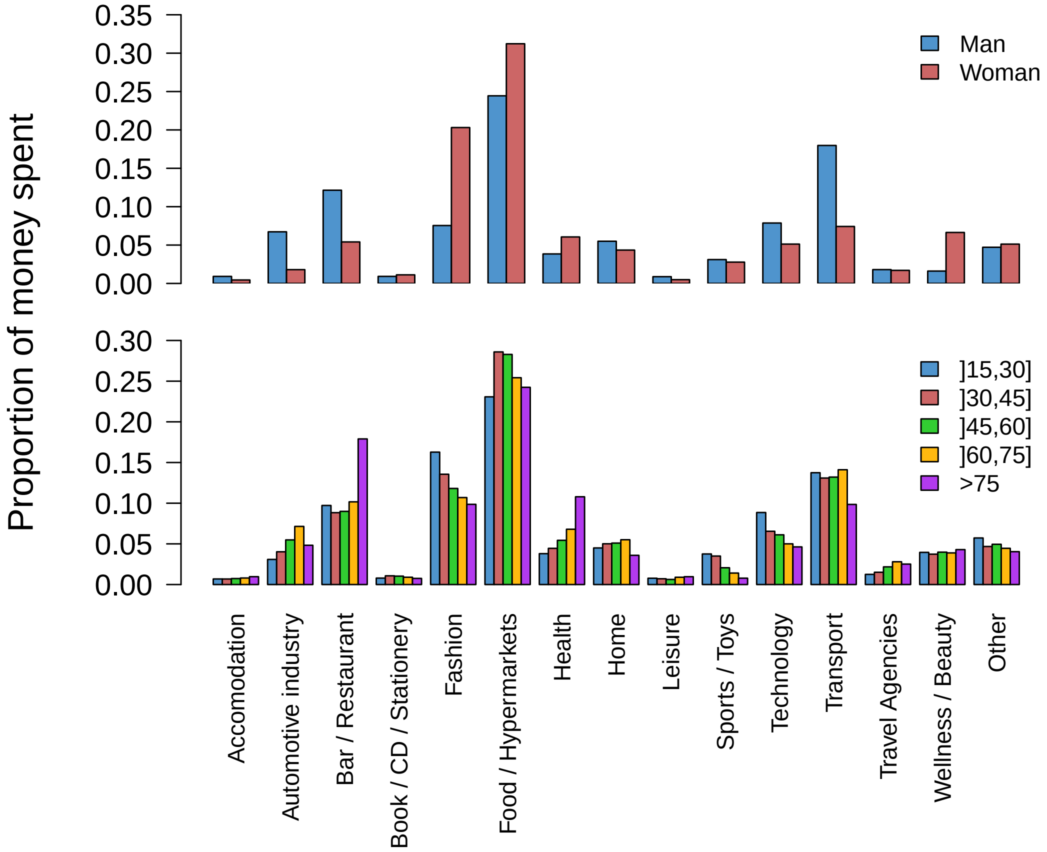

Figure S7: **Average fraction of money spent by an employed individual according to the business category and to his/her gender and age.**

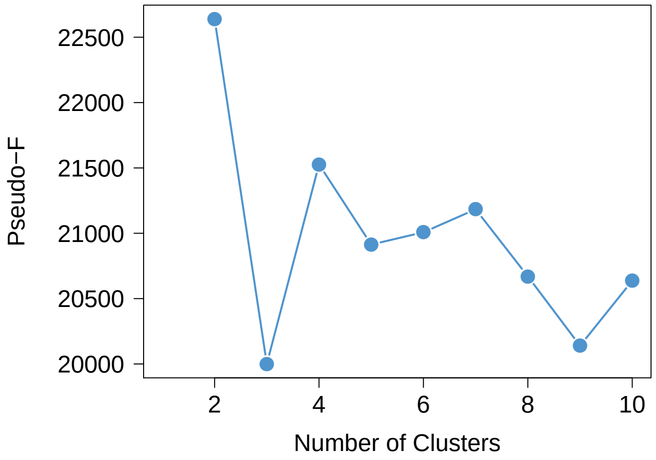

Figure S8: **Pseudo-F as a function of the number of clusters.** K-means clustering algorithm with Euclidean distance applied on the normalized distributions of money spent according to the hour of the day.

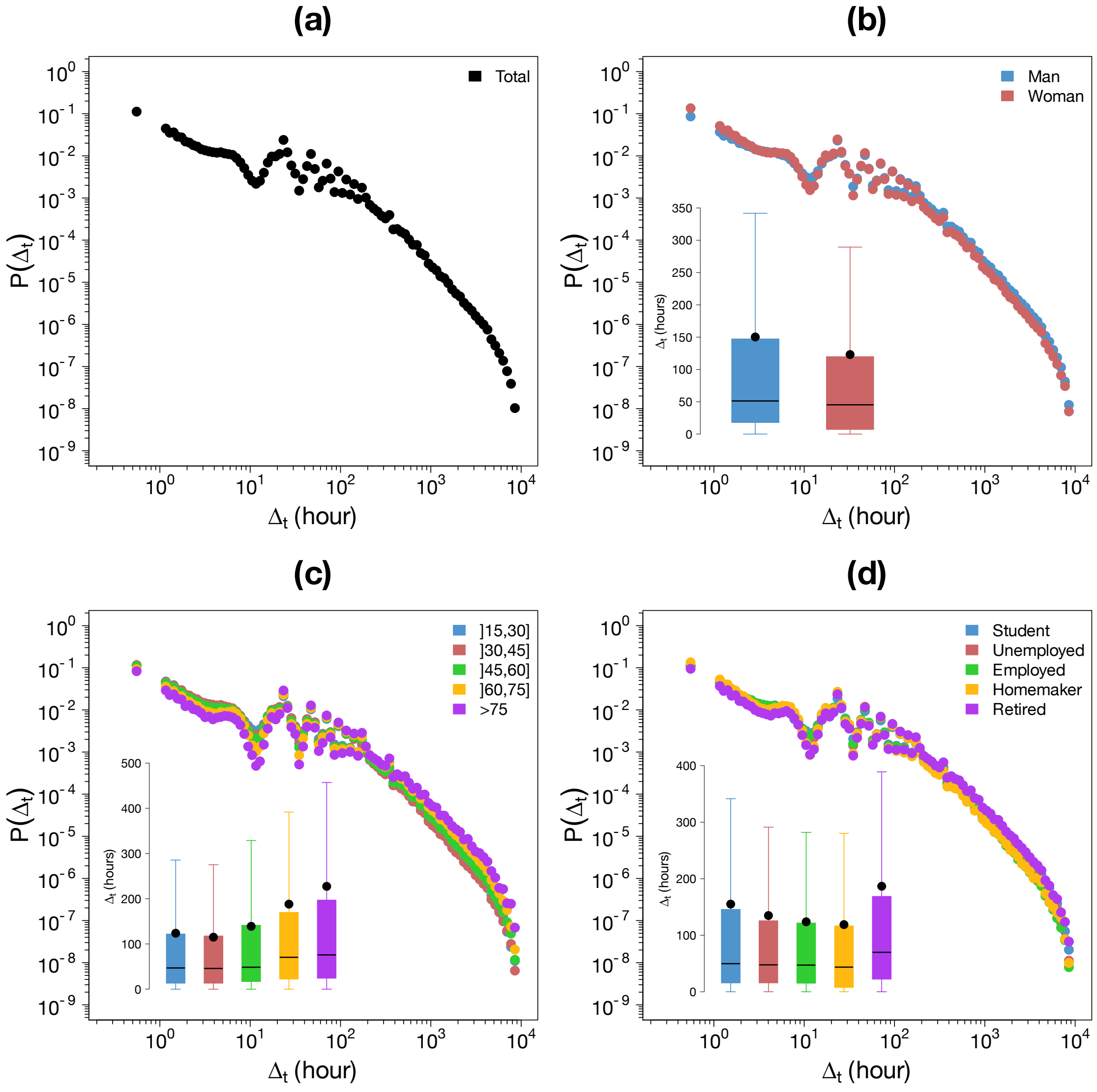

Figure S9: **Inter-event time distribution** $P(\Delta_t)$**.** (a) Probability density function of Δ_t. (b) - (d) Probability density function of Δ_t according to the gender (b), the age (c) and the occupation (d). The insets show the Tukey boxplot of the distributions, the black points represent the average.

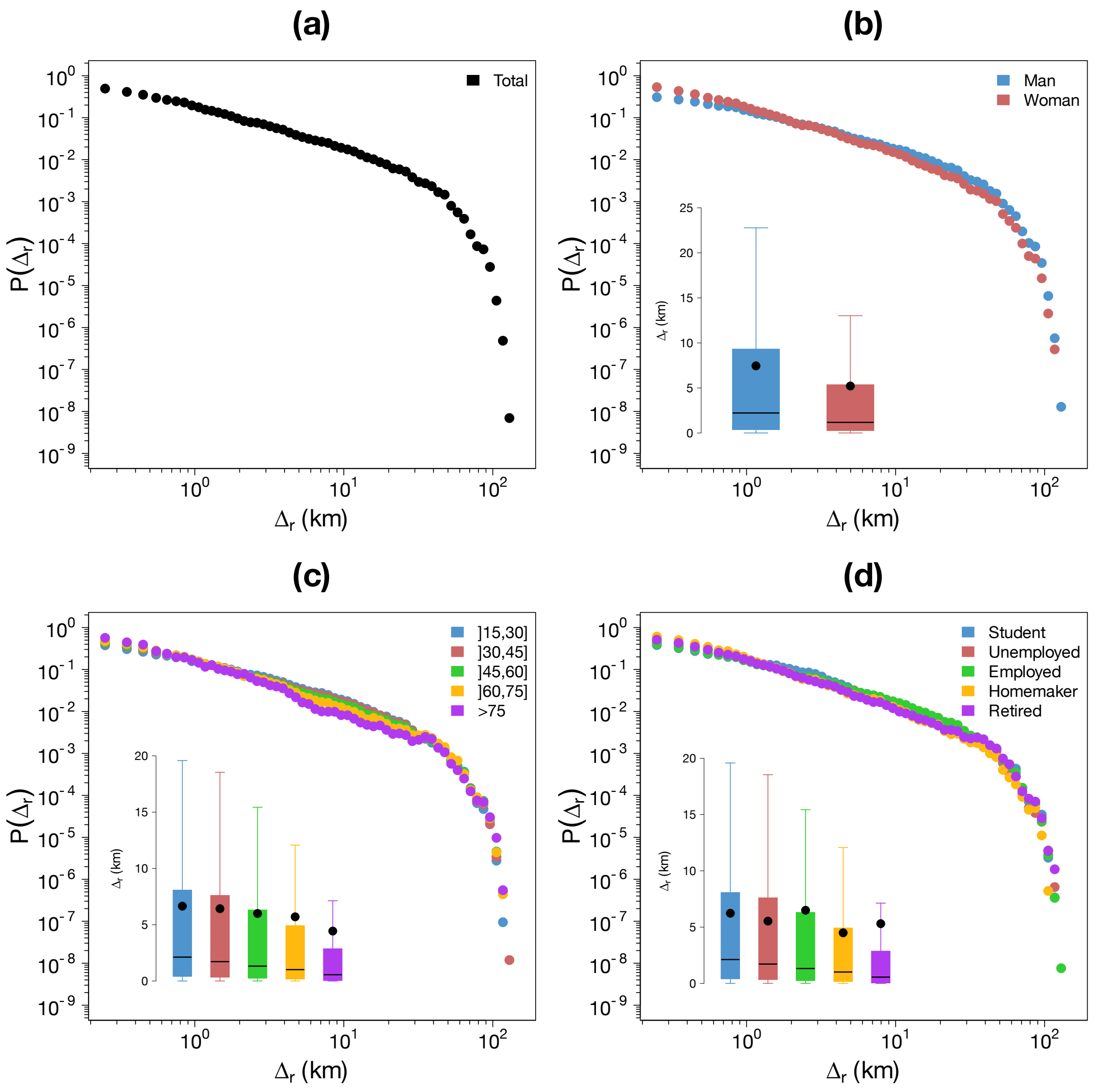

Figure S10: **Distribution of the distance traveled by a customer between two consecutive transactions** $P(\Delta_r)$**.** (a) Probability density function of Δ_r. (b) - (d) Probability density function of Δ_r according to the gender (b), the age (c) and the occupation (d). The insets show the Tukey boxplot of the distributions, the black points represent the average.

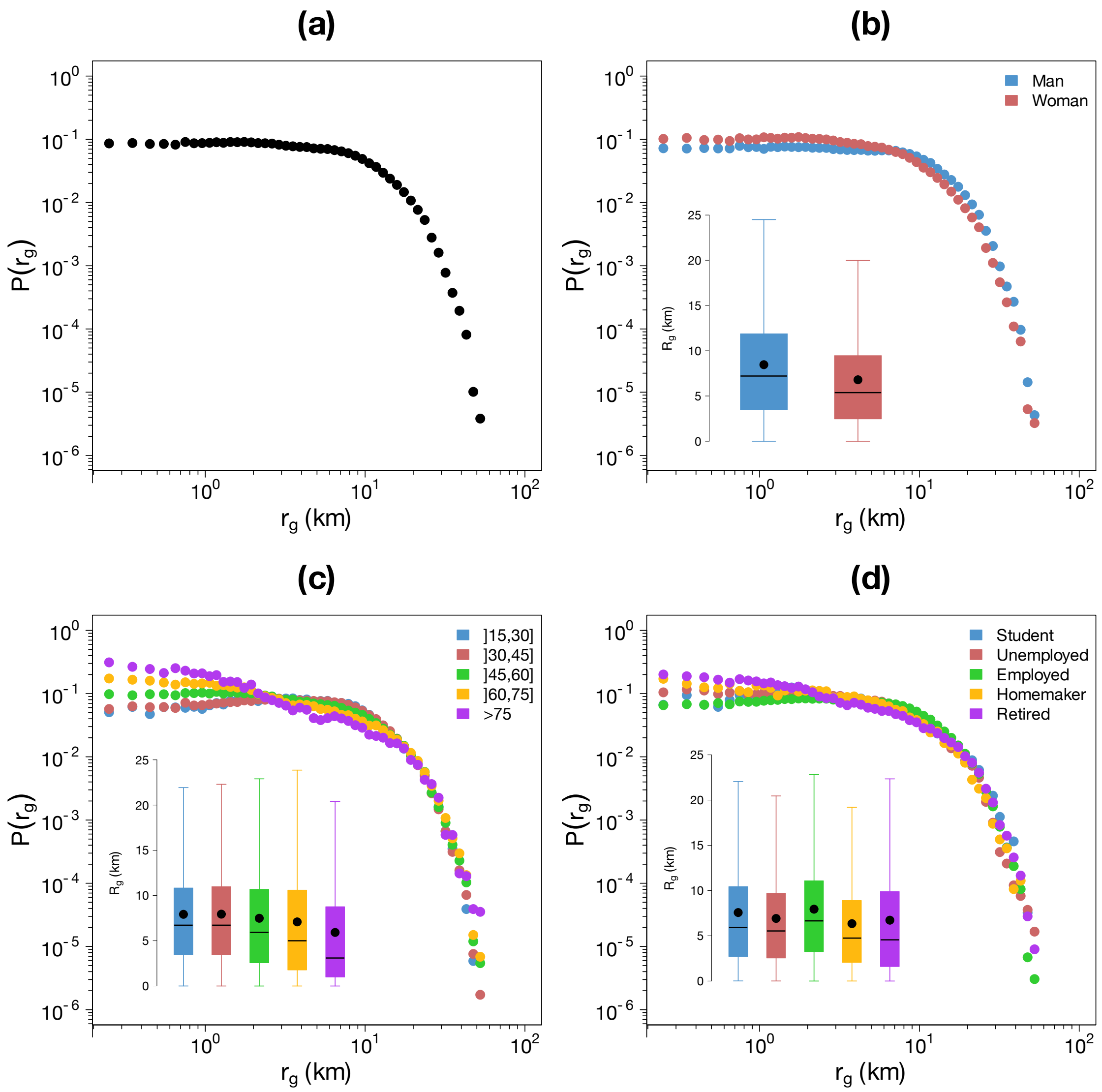

Figure S11: **Distribution of the radius of gyration $P(r_g)$.** (a) Probability density function of r_g. (b) - (d) Probability density function of r_g according to the gender (b), the age (c) and the occupation (d). The insets show the Tukey boxplot of the distributions, the black points represent the average.

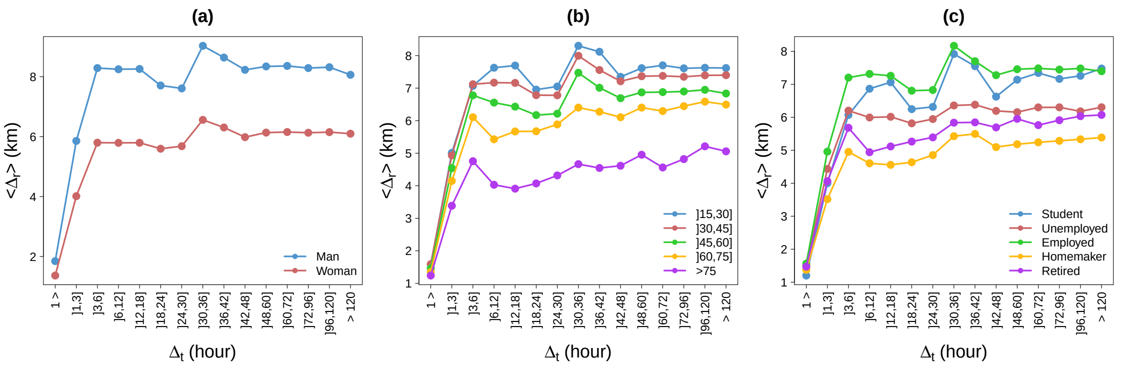

Figure S12: **Average $< r_g >$ value as a function of Δ_t according to the gender (a), the age (b) and the occupation (c).**

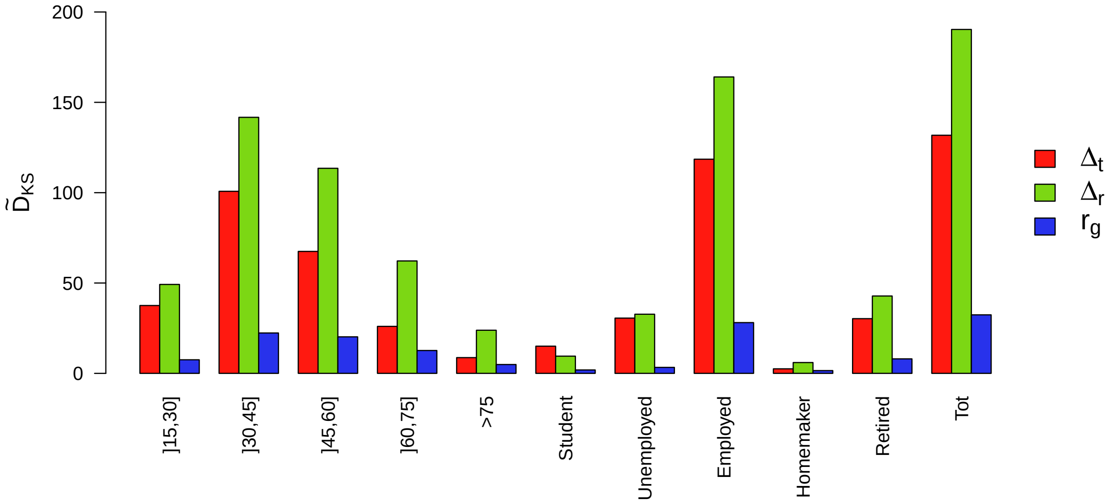

Figure S13: **Kolmogorov-Smirnov distance between men and women's Δ_t distributions (in red), Δ_r distributions (in green) and r_g distributions (in blue) according to their demographic characteristics.**

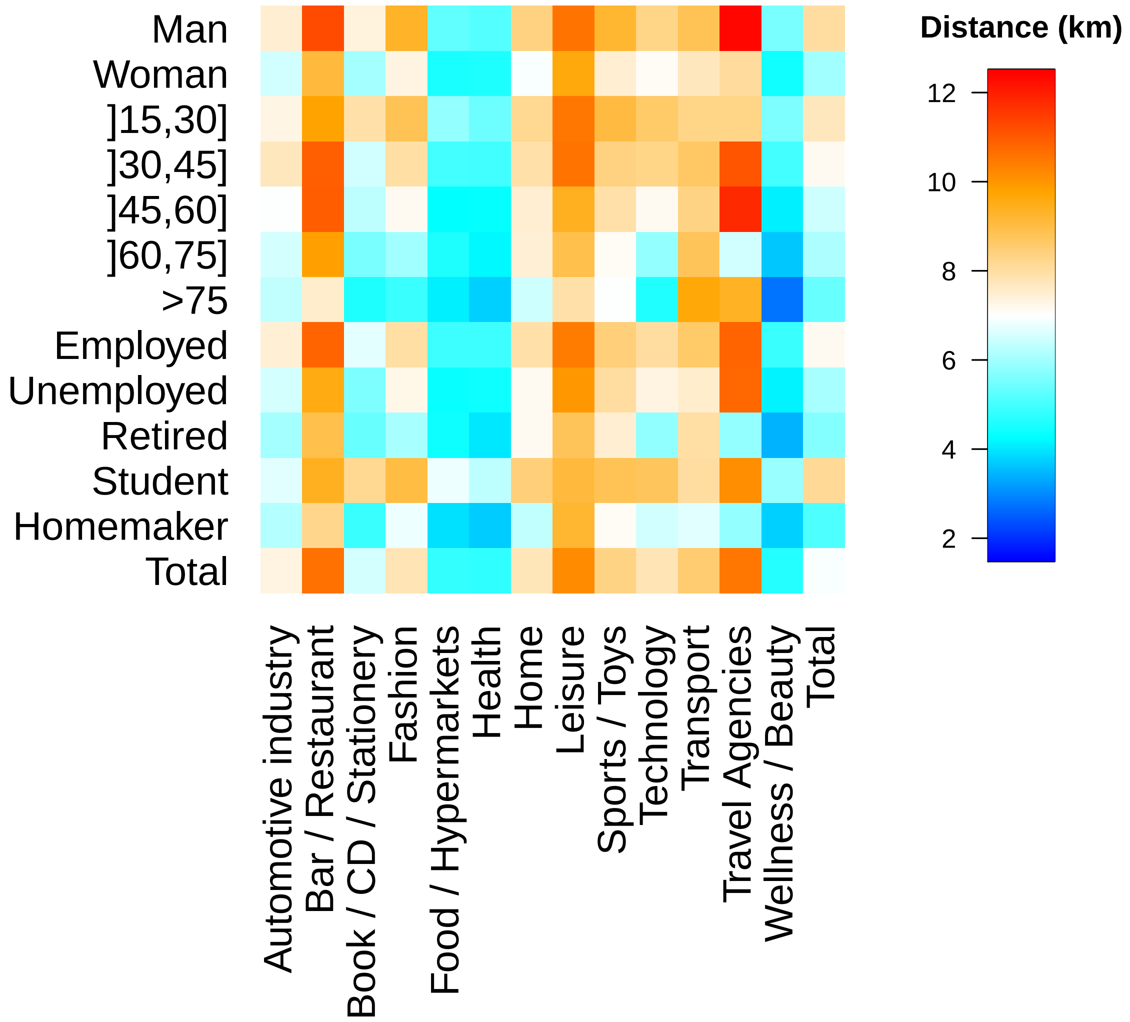

Figure S14: **Average distance between individuals' place of residence and business according to individual's demographics and business' category.** Distances are expressed in kilometer and are computed using the Haversine distance between the latitude and longitude coordinate of the centroid of the customer's postcode of residence and the business' latitude and longitude coordinates.

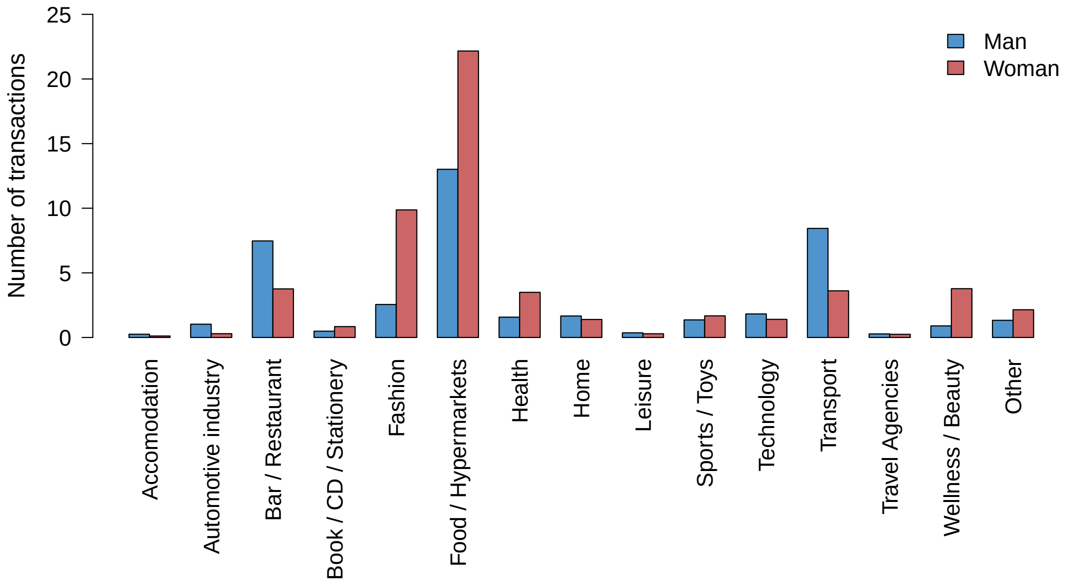

Figure S15: **Average number of transactions according to the gender and the business category.**
